# Robustification of Linear Regression and Its Application in Genome-Wide Association Studies

**DOI:** 10.3389/fgene.2020.00549

**Published:** 2020-06-08

**Authors:** Md. Alamin, Most. Humaira Sultana, Haiming Xu, Md. Nurul Haque Mollah

**Affiliations:** ^1^Institute of Crop Science and Institute of Bioinformatics, College of Agriculture and Biotechnology, Zhejiang University, Hangzhou, China; ^2^Bioinformatics Lab, Department of Statistics, University of Rajshahi, Rajshahi, Bangladesh

**Keywords:** linear regression, robustness, β-divergence method, β-selection, β-test statistic, permutation test, GWAS, genes and SNPs

## Abstract

Regression analysis is one of the most popular statistical techniques that attempt to explore the relationships between a response (dependent) variable and one or more explanatory (independent) variables. To test the overall significance of regression, F-statistic is used if the parameters are estimated by the least-squares estimators (LSEs), while if the parameters are estimated by the maximum likelihood estimators (MLEs), the likelihood ratio test (LRT) statistic is used. However, both procedures produce misleading results and often fail to provide good fits to the reasonable space of the dataset in the presence of outlying observations. Moreover, outliers occur very frequently in any real datasets as well as in the molecular OMICS datasets. Hence, an effort is made in this study to robustify MLE based regression analysis by maximizing the β-likelihood function. The tuning parameter β is selected by cross-validation. For β = 0, the proposed method reduces to the classical MLE based regression analysis. We inspect the performance of the proposed method using both synthetic and real data analysis. The results of simulations indicate that the proposed method performs better than traditional methods in both outliers and high leverage points to estimate the parameters and mean square errors. The results of relative efficiency analysis show that the proposed estimator is relatively less affected than the popular estimators, including S, MM, and fast-S for normal error distribution in case high dimension and outliers. Also, real data analysis results demonstrated that the proposed method shows robust properties with respect to data contaminations, overcome the drawback of the traditional methods. Genome-wide association studies (GWAS) by the proposed method identify the vital gene influencing hypertension and iron level in the liver and spleen of mice. Furthermore, we have identified 15 and 21 significant SNPs for chalkiness degree and chalkiness percentage, respectively, by GWAS based on the proposed method. The variant of the SNPs might be provided the new resources for grain quality traits and could be used for further molecular and physiological analysis to enhance the better quality of rice grain. These results offer an important basis for further understanding of the robust regression analysis, which might be applied in various fields, including business, genetics, and bioinformatics.

## Introduction

Regression is the mathematical measure for the average relationship between two or more variables in terms of the original units at the data. It is a popular statistical technique for predicting the response variable for fixed value (s) of the independent variable (s). The purpose of linear regression analysis is to fit the straight line to the observed data. It is widely used in multi-disciplinary research areas. Let us consider a *p* variables linear regression model as follows:

(1) $$\begin{array}{l} y_{i}=\gamma_{0}+\overset{p}{\underset{j=1}{\sum }}\gamma_{j}x_{ij}+u_{i} \end{array}$$

where *y*_*i*_ (*i* = 1, 2, …, *n*) is the *i*th observation of the response variable; γ_0_ is the intercept term; γ_*j*_ (*j* = 1, 2, …, *p*) are the regression parameters; *x*_*ij*_ (*j* = 1, 2, …, *p*) are the *i*th observation of *p* explanatory variables that are assumed to be independent of each other and *u*_*i*_ is the random error term corresponding to *i*th observation. We assumed that *u*_*i*_ follows normally and identically distributed with mean zero and variance σ^2^. Under this assumption, the density function of *y*_*i*_ is given by:

(2) $$\begin{array}{l} f_{\theta }\left(y_{i}\right)={\left(2\pi \sigma^{2}\right)}^{{-}^{1}{/}_{2}}\exp\left\{-\frac{1}{2\sigma^{2}}{\left(y_{i}-\gamma_{0}-\overset{p}{\underset{j=1}{\sum }}\gamma_{j}x_{ij}\right)}^{2}\right\} \end{array}$$

and the average log-likelihood function for the model parameter $\theta =\left(\gamma_{0},\gamma_{1},\ldots ,\gamma_{p},\sigma^{2}\right)$ in Equation (2) is given by:

(3) $$\begin{array}{l} \begin{array}{c} \bar{L}\left(\theta |Y,X\right)=\frac{1}{n}\overset{n}{\underset{i=1}{\sum }}\log f_{\theta }\left(y_{i}\right)=\frac{1}{n}\log L\left(\theta |Y,X\right)\\ =-\frac{1}{2n}\log\left(2\pi \sigma^{2}\right)-\frac{1}{2n\sigma^{2}}{\left(Y-X\gamma \right)}^{\prime }\left(Y-X\gamma \right) \end{array} \end{array}$$

where $\gamma ={\left(\gamma_{0},\gamma_{1},\ldots ,\gamma_{p}\right)}^{\prime }$, $Y={\left(y_{1},y_{2},\ldots ,y_{n}\right)}^{\prime }$ and *X* = [*x*_*ij*_] is the matrix of order *n* × (*p* + 1) with *x*_*i*0_ = 1 *for i* = 1, 2, …, *n*. Here the problem is to test the significance of the regression parameters with the following hypothesis:

(4) $$\begin{array}{l} H_{0}:\;\gamma_{1}=\gamma_{2}\ldots =\gamma_{p}\text{ vs}.\;H_{1}\;:\;H_{0}\text{ is not true} \end{array}$$

To test the null hypothesis (*H*_0_) against the alternative hypothesis (*H*_1_), the F-statistic is used if the parameters are estimated by the least-squares estimators (LSEs). On the other hand, if the parameters are estimated by the maximum likelihood estimators (MLEs), the likelihood ratio test (LRT) statistic is used to test the null hypothesis. Because of our interest in the improvement of classical MLE based regression analysis, let us first introduce LRT statistic as follows:

(5) $$\begin{array}{l} \chi_{0}^{2}=-2\log\lambda =-2\log\frac{L\left({\tilde{\theta }}_{0}|Y\right)}{L\left({\hat{\theta }}_{1}|Y\right)}=2n\left\{L\left({\hat{\theta }}_{1}|Y\right)-L\left({\tilde{\theta }}_{0}|Y\right)\right\}\\ \;=\log\left({\tilde{\sigma }}^{2}/{\hat{\sigma }}^{2}\right) \end{array}$$

which follows ~χ^2^−distribution with 1 degree of freedom, where ${\tilde{\theta }}_{0}=\left({\tilde{\gamma }}_{0},\;{\tilde{\sigma }}^{2}\right)$ and ${\hat{\theta }}_{1}=\left(\hat{\gamma },\;{\hat{\sigma }}^{2}\right)$ with $\hat{\gamma }=\left({\hat{\gamma }}_{0},\;{\hat{\gamma }}_{1},\;\ldots ,\;{\hat{\gamma }}_{p}\right)$ are the MLEs of $\theta_{0}=\left(\gamma_{0},\;\sigma^{2}\right)$ and $\theta_{1}=\left(\gamma ,\;\sigma^{2}\right)$ with γ = (γ_0_, γ_1_,…, γ_*p*_) under *H*_0_ and *H*_1_, respectively. Here:

(6) $$\begin{array}{l} {\tilde{\gamma }}_{0}=ȳ, \end{array}$$

(7) $$\begin{array}{l} {\tilde{\sigma }}^{2}=\frac{1}{n}\overset{n}{\underset{i=1}{\sum }}{\left(y_{i}-{\tilde{\gamma }}_{0}\right)}^{2}=\frac{1}{n}{\left(Y-\gamma_{0}1_{1}\right)}^{\prime }\left(Y-\gamma_{0}1_{1}\right), \end{array}$$

(8) $$\begin{array}{l} \hat{\gamma }={\left(X^{\prime }X\right)}^{-1}X^{\prime }Y\text{,} \end{array}$$

(9) $$\begin{array}{l} \text{and }{\hat{\sigma }}^{2}=\frac{1}{n}{\left(Y-X\hat{\gamma }\right)}^{\prime }\left(Y-X\hat{\gamma }\right) \end{array}$$

Note that the least squares estimator (LSE) and maximum likelihood estimator (MLE) of γ are exactly the same, but not for σ^2^. The LSE of σ^2^ has divisor (*n - p*) whereas the MLE of σ^2^ has divisor *n* instead of (*n–p*). However, it is well-known that both classical LSE and MLE for the regression parameters are very much sensitive to outliers. So, regression analysis by anyone of classical LSE and MLE produces misleading results in the presence of outlying observations.

To overcome this problem, there exists several robust regression approaches like M-Huber, M-Hampel, M-Tukey, LTS, LMS, MM, S, fast-S, and so on in the literature. The major role of robust regression analysis is to provide an excellent fit to the reasonable space of the data. Generally, efficiency and breakdown point is used to investigate the performance of robust techniques. Efficiency can tell us how well-robust techniques perform relative to least squares on clean data. High efficiency is mostly desired on estimation. The breakdown point is a measure for the efficiency of the estimator when the sample contains a significant fraction of outliers (Hampel, 1975). Some robust regression estimates asymptotic breakdown point is a maximum of 50%. Rousseeuw (1984) introduced the LMS (least median of squares) and least trimmed squares (LTS) estimators for robust estimation. Rousseeuw and Yohai (1984) introduced S-estimator, which is an equivariant estimator for linear regression with asymptotic breakdown point 0.5. MM-estimates (Yohai, 1987) and the τ-estimates (Yohai and Zamar, 1988) are also the more efficient estimates with asymptotic breakdown point 0.5. A modification of the subsampling algorithm for the LTS estimator was proposed by Rousseeuw and Van Driessen (2006), which is called fast-LTS that considerably improves its performance. Moreover, the fast-LTS estimator is much faster than the approximating algorithm for the LTS estimator. Recently, Salibian-Barrera and Yohai (2006) proposed a fast-S algorithm using Rupperts SURREAL algorithm (Ruppert, 1992) similar to the fast-LTS of Rousseeuw and Van Driessen (2006). However, most of the robust approaches have some drawbacks. For example, the robustness performance of those approaches gradually becomes weaker if the number of explanatory variables increases in the linear regression model (Rousseeuw and Yohai, 1984; Salibian-Barrera and Yohai, 2006).

Therefore, in this paper, a new robust regression method by maximizing the β-likelihood function is proposed, and its performance is compared with the existing approaches including LS, M-Huber (Huber, 1973), M-Hampel (Hampel, 1974), M-Tukey (Beaton and Tukey, 1974), LTS, LMS, MM, S, and as well as fast-S (Salibian-Barrera and Yohai, 2006). Simulation and real data analysis were carried out in this study to inspect the performance of the proposed method. Mean square error (MSE) was also estimated to investigate the performance of the proposed methods when the number of explanatory variables is increased in the regression model with the absence and presence of outliers. Also, relative efficiency analysis was carried out to check the efficiency of the proposed estimator compares with other popular estimators such as S, MM, and fast-S for normal error distribution in case high dimension and outliers in this study. Moreover, real data analysis was performed to confirm the performance of the proposed method. Furthermore, we have applied GWAS for testing the performance of our proposed method for the identification of important genes/SNPs related to grain quality in rice.

## Methods and Materials

### Robustification of MLE Based Linear Regression Analysis by Maximizing β-Likelihood Function

The β-likelihood function for the model parameter $\theta =\left(\gamma_{0},\gamma_{1},\ldots ,\gamma_{p},\sigma^{2}\right)$ of Equation (2) is defined as:

(10) $$\begin{array}{l} L_{\beta }\left(\theta |Y\right)=\frac{1}{\beta }\left\{\frac{1}{nl_{\beta }\left(\theta \right)}\overset{n}{\underset{i=1}{\sum }}f_{\theta }^{\beta }\left(y_{i}\right)-1\right\} \end{array}$$

where

(11) $$\begin{array}{l} l_{\beta }\left(\theta \right)={\left\{\int f_{{\;}_{\theta }}^{1+\beta }\left(y\right)dy\right\}}^{{\;}^{\beta }{/}_{1+\beta }}={\left(1+\beta \right)}^{{-}^{\beta }{/}_{2\left(1+\beta \right)}}\\ \;{\left(2\pi \sigma^{2}\right)}^{{-}^{\beta^{2}}{/}_{2\left(1+\beta \right)}}. \end{array}$$

We call *L*_β_ (**θ** *| Y)* in Equation (10) as the β-likelihood function, since it reduces to the average of log-likelihood function (3) for β → 0. The β-likelihood function (10) is induced from the β-divergence (Mollah et al., 2010). This divergence is also known as density power divergence (Basu et al., 1998; Mihoko and Eguchi, 2002). It measures the discrepancy between two density functions. The minimizer of β-divergence is equivalent to maximizer of β-likelihood function. To robustify linear regression analysis, we estimate the model parameter vector **θ** by maximizing β-likelihood function under hypotheses *H*_0_ and *H*_1_, respectively. To test *H*_0_ against *H*_1_ as defined in equation (4), the proposed test statistic (criterion) is defined by:

(12) $$\begin{array}{l} \chi_{\beta }^{2}=2n\left\{L_{\beta }\left({\hat{\theta }}_{1\beta }\right)-L_{\beta }\left({\tilde{\theta }}_{0\beta }\right)\right\}, \end{array}$$

which we call β-test statistic, where ${\tilde{\theta }}_{0\beta }=\left({\tilde{\gamma }}_{0\beta },{\tilde{\sigma }}_{\beta }^{2}\right)$ and ${\hat{\theta }}_{1\beta }=\left({\hat{\gamma }}_{\beta },{\hat{\sigma }}_{\beta }^{2}\right)$ with ${\hat{\gamma }}_{\beta }=\left({\hat{\gamma }}_{0\beta },{\hat{\gamma }}_{1\beta },\ldots ,{\hat{\gamma }}_{p\beta }\right)$ are the maximum β-likelihood estimators (β-MLE) of $\theta_{0}=\left(\gamma_{0},\sigma^{2}\right)$ and $\theta_{1}=\left(\gamma ,\sigma^{2}\right)$ with γ = (γ_0_, γ_1_, …, γ_*p*_) under *H*_0_ and *H*_1_, respectively. Under *H*_0_, the estimates ${\tilde{\theta }}_{0\beta }=\left({\tilde{\gamma }}_{0\beta },{\tilde{\sigma }}_{\beta }^{2}\right)$ of $\theta_{0}=\left(\gamma_{0},\sigma^{2}\right)$ are obtained iteratively as follow:

(13) $$\begin{array}{l} {\tilde{\gamma }}_{0\beta ,t+1}=\frac{\overset{n}{\underset{i=1}{\sum }}w_{\beta }\left(y_{i}|{\tilde{\theta }}_{0\beta ,t}\right)y_{i}}{\overset{n}{\underset{i=1}{\sum }}w_{\beta }\left(y_{i}|{\tilde{\theta }}_{0\beta ,t}\right)}={W^{\prime }}_{0\beta ,t}Y{\left({W^{\prime }}_{0\beta ,t}1\right)}^{-1}, \end{array}$$

(14) $$\begin{array}{l} {\tilde{\sigma }}_{{\;}_{\beta ,t+1}}^{2}=\frac{\overset{n}{\underset{i=1}{\sum }}w_{\beta }\left(y_{i}|{\tilde{\theta }}_{0\beta ,t}\right){\left(y_{i}-{\tilde{\gamma }}_{0\beta ,t}\right)}^{2}}{\overset{n}{\underset{i=1}{\sum }}w_{\beta }\left(y_{i}|{\tilde{\theta }}_{0\beta ,t}\right)}\\ \;={W^{\prime }}_{0\beta ,t}\left[\left(Y-{\tilde{\gamma }}_{0\beta ,t}1\right)\#\left(Y-{\tilde{\gamma }}_{0\beta ,t}1\right)\right]{\left({W^{\prime }}_{0\beta ,t}1\right)}^{-1} \end{array}$$

where $W_{0\beta }={\left[w_{\beta }\left(y_{i}|\theta_{0}\right)\right]}_{n\times 1}\;\text{and}1\;=\text{(1, 1,}\ldots {\text{, 1)}}^{\prime }\text{.}$ Here we call:

(15) $$\begin{array}{l} w_{\beta }\left(y_{i}|\theta_{0}\right)=\exp\left\{-\frac{\beta }{2\sigma^{2}}{\left(y_{i}-\gamma_{0}\right)}^{2}\right\} \end{array}$$

as the β-weight function under *H*_0_. It produces smaller weights for outlying/contaminated observations. The notation “#” denotes the Hadamard product.

Under *H*_1_, the estimate ${\hat{\theta }}_{1\beta }=\left({\hat{\gamma }}_{\beta },{\hat{\sigma }}_{\beta }^{2}\right)$ of $\theta_{1}=\left(\gamma ,\sigma^{2}\right)$ are obtained iteratively as follows:

(16) $$\begin{array}{l} {\hat{\gamma }}_{\beta ,t+1}={\left(X^{\prime }X_{\beta ,t}\right)}^{-1}{X^{\prime }}_{\beta ,t}Y, \end{array}$$

(17) $$\begin{array}{l} {{\hat{\sigma }}^{2}}_{\beta ,t+1}={W^{\prime }}_{1\beta ,t}\left[\left(Y-X{\hat{\gamma }}_{\beta ,t}\right)\#\left(Y-{\hat{\gamma }}_{\beta ,t}\right)\right]{\left({W^{\prime }}_{1\beta ,t}1\right)}^{-1}, \end{array}$$

where $X_{\beta }={\left[X\#W_{1\beta }{1^{\prime }}_{1}\right]}_{n\times \left(p+1\right)},1_{1}={\left(1,1,\ldots ,1\right)}^{\prime }\;\text{and}\;W_{1\beta }={\left[w_{\beta }\left(y_{i}|\theta_{1}\right)\right]}_{n\times 1}$.

Here $w_{\beta }\left(y_{i}|\theta_{1},X\right)=\exp\left\{-\frac{\beta }{2\sigma^{2}}{\left(y_{i}-\gamma_{0}-\overset{p}{\underset{j=1}{\sum }}\gamma_{j}x_{ij}\right)}^{2}\right\}$ is the β-weight function under *H*_1_. This weight function produces smaller weights for outlying/contaminated observations. The proposed test statistic (12) reduces to the classical MLE based test statistic (5).

### Hypothesis Testing

To test the null hypothesis (*H*_0_) against the alternative hypothesis (*H*_1_) as defined in (4) from the robustness point of view, we can compute *p*-values using asymptotic χ^2^ distribution as defined in (5) by replacing ${\tilde{\sigma }}^{2}$ and ${\hat{\sigma }}^{2}$ by the proposed estimates ${\tilde{\sigma }}_{\beta }^{2}$ and ${\hat{\sigma }}_{\beta }^{2}$, respectively. However, we propose to use permutation-based *p*-values to test whether *H*_0_ is true or false. To compute permutation-based *p*-values, first we compute the value of $\chi_{\beta }^{2}$ as defined in (12) based on the original dataset. Then we permute the values of the response variable *N* times and each time we compute $\chi_{\beta }^{2}$. Then we compute the *p*-values (*p*) for testing *H*_0_ against *H*_1_ using the following formula:

(18) $$\begin{array}{l} p=\overset{N}{\underset{k=1}{\sum }}I_{[{{\hat{\chi }}_{\beta }}^{2}{\;}_{\left(k\right)}\leq {{\hat{\chi }}_{\beta }}^{2}]}/N, \end{array}$$

where ${\hat{\chi }}_{\beta }^{2}$denotes the estimate of $\chi_{\beta }^{2}$ with the original dataset and ${\hat{\chi }}_{\beta }^{2}\left(k\right)$ denotes the estimate of $\chi_{\beta }^{2}$ with the *k*th permuted values of the response variable in the dataset. Note that, for β → 0, $\chi_{\beta }^{2}$ reduces to the approximate χ^2^distribution.

### Robustness of the Estimators

Suppose *G* be the distribution function of g, and then we can view the β-estimator (θ_β_) of θ as an M-estimator, which is a function of G defined by:

(19) $$\begin{array}{l} \theta_{\beta }\left[G\right]=\underset{\theta }{\text{argmax }}\left\{\int \Omega_{\beta }\left(y;\theta \right)dG\left(y\right)\right\} \end{array}$$

where $\Omega_{\beta }\left(y;\theta \right)=\left[\frac{1}{\beta l_{\beta }\left(\theta \right)}f_{\theta }^{\beta }\left(y\right)-1\right]$ and $f_{\theta }\left(y\right)=N\left(\gamma_{0}+\overset{p}{\underset{j=1}{\sum }}\gamma_{j}x_{ij},\sigma^{2}\right)$. Then the influence function (IF) for the β-estimator at *y* under the distribution function *G* is defined as:

(20) $$\begin{array}{l} \text{IF }\left(y;\theta_{\beta },G\right)=\underset{\;\varepsilon \;\to \text{0}}{\text{lim}}\left\{\theta_{\beta }\left[\left(1-\varepsilon \right)G+\varepsilon \Delta_{y}\right]-\theta_{\beta }\left[G\right]\right\}/\varepsilon \end{array}$$

where, Δ_*y*_ is the probability measure that puts mass one at the point *y*. An estimator is supposed to be B-robust if its influence function is a bounded function of *y* (Hampel et al., 1986). Since the β-estimator fulfills the properties of M-estimator, the influence function for the proposed estimator also can be written as:

(21) $$\begin{array}{l} \text{IF }\left(y;\theta_{\beta },G\right)=H{\left({\text{Ψ}}_{\beta },G\right)}^{-1}{\text{Ψ}}_{\beta }\left(y;\theta_{\beta }\left[G\right]\right) \end{array}$$

where Ψ_β_ (*y*; θ) = δΩ_β_ (*y*; θ)/δθ is the estimating function for the β**-**estimator and

(22) $$\begin{array}{l} H\left({\text{Ψ}}_{\beta },G\right)=-\int {\left[\frac{\delta {\text{Ψ}}_{\beta }\left(y;\theta \right)}{\delta \theta }\right]}_{\theta =\theta \left[G\right]}dG\left(y\right) \end{array}$$

is a matrix which does not rely on y; thus, the B-robustness is equivalent to the boundedness of the estimating function for the M-estimator as well as the β**-**estimator (Mihoko and Eguchi, 2002). To show the finiteness of the estimating function Ψ_β_(*y*; θ) for the β-estimator, let us consider the general form of two variables linear regression models for estimating function as defined by:

(23) $$\begin{array}{l} {\text{Ψ}}_{\beta }\left(y;\theta \right)=\left[f_{\theta }^{\beta }\left(y\right)\times \frac{\delta {\left[l_{\beta }\left(\theta \right)\right]}^{-1}}{\delta \theta }\right]\\ \;+\left[{\left[l_{\beta }\left(\theta \right)\right]}^{-1}f_{\theta }^{\beta }\left(y\right)\frac{\delta \log\left[f_{\theta }\left(y\right)\right]}{\delta \theta }\right] \end{array}$$

Obviously, in the estimating function (23), (i) *l*_β_(θ) is independent on observation (ii) $f_{\theta }^{\beta }\left(y\right)\to 0$ for β > 0 and *y* → ∞, and (iii) log[ *f*_θ_(*y*)] → ∞ for *y* → ∞. Thus, we observe that the boundedness of the estimating function depends only on the second term of the right-hand side of equation (23). We also observe that second term will be bounded for any *y* when β > 0. Thus, we can conclude that all components of estimating function (23) are bounded to any *y* when β > 0. Therefore, our proposed β-estimators are B-robust against outliers. However, the robustness performance of the proposed method depends on the value of the tuning parameter β and the initialization of the regressions parameters.

To obtain high-breakdown estimators with 50% breakdown points using Equations (13–17), we are proposing the following procedure for initialization of the regression parameters. Let $D=\;\left\{z_{i}|z_{i}=\left(y_{i},\;x_{i}\right),\;i=1,\;2,\ldots ,\;n\right\}\;$be the entire data space, where *y*_*i*_is the *i*th component of ***Y*** and **x**_*i*_ is the *i*th row of ***X*** as defined in Equation (3). Let us define a subset $D_{o}\subset D$ based on Euclidean distance as follows:

(24) $$\begin{array}{l} D_{o}=\;\left\{z_{i}\;\in D|d_{i}=∥z_{i}-\;z_{md}∥\;<\xi_{p}\;i=1,\;2,\ldots ,\;n\right\} \end{array}$$

where ξ_*p*_ is the *p*th percentile of {*d*_1_, *d*_2_,…, *d*_*n*_} with *p* = 50. The notation ${‡}_{md}$ is the median vector as defined by:

(25) $$\begin{array}{l} z_{\text{md}}={\left(\underset{i=1,2,\ldots ,n}{\text{median}}\left(y_{i}\right),\underset{i=1,2,\ldots ,n}{\text{median}}\left(x_{1i}\right),\ldots ,\underset{i=1,2,\ldots ,n}{\text{median}}\left(x_{pi}\right)\right)}^{T} \end{array}$$

Then the appropriate initial values of **θ** for the proposed estimators (13–14) and (16–17) can be obtained by the classical estimators (6–7) and (8–9), respectively, using the reduced dataset${\;D}_{o}$. The proposed estimators of **θ** can resist the effect of 50% breakdown points if we compute the initial values of the location parameter vector μ and scatter matrix *V* by a good part of the dataset. If the initial value of **θ** close to the mean vector **μ**_*i*_, then β-weight function produces larger weights (close to 1) for each data vector of the data cluster having mean the vector μ_*i*_ and the covariance matrix *Vi* and smaller weights (close to 0) for the data vectors of the other clusters, respectively. Thus, iterative Equations 16–17 can produce $\left({\hat{\mu }}_{i,\;\beta },\;{\hat{V}}_{i,\;\beta }\right)\;$for (μ_*i*_, *V*_*i*_) considering data vectors in other clusters as outliers. Similarly, other pairs of mean vector and covariance matrix can be estimated sequentially. A detailed discussion can be found in Mollah et al. (2010).

Breakdown points of the proposed estimates depend on the value of the tuning parameter β and initialization of the parameters in Equations 13–14. However, both factors work through the β-weight function as defined in Equation 15. To discuss in detail, let us rewrite the β-weight function as follows, which lies between 0 and 1. It produces a smaller weight (≥ 0) if the data vector (***x***) is unusual or contaminated by outliers and the more substantial weight (≤ 1) if ***x*** is usual/uncontaminated. Generally, a data vector (***x***) is known as unusual/contaminated if it is far away from the mean vector **μ**. The β-weight function is the negative exponential function of the squared Mahalanobis Distance (MD) defined by *MD* = (*x* − μ)′*V*^−1^(*x* − μ) ≥ 0 between the data vector ***x*** and the mean vector **μ**. From equation 15, it is seen that the β-weight decreases (increases) when MD increases (decreases). Estimates become robust by putting the smaller weight corresponding to the unusual/contaminated data vector and larger weight corresponding to the usual/uncontaminated data vectors in Equations 16–17.

### Selection of the Tuning Parameter β

The value of β acts a vital role in the performance of the proposed method. A larger β increases the robustness but decreases the efficiency of estimators and vice-versa for the smaller β. Therefore, an appropriate value of β may control the trade-off between the robustness and efficiency of estimators. To find an appropriate β for the maximum β-likelihood estimators used the negative of the β-likelihood function (10) with a defined value β_0_ of β as a measure for evaluation of the β-estimators. In this study, we also use the same measure for β selection using cross-validation (Friedman et al., 2001). To determine the measure for β selection using *K*-fold cross-validation, let us split the entire dataset $D=\;\left\{\left(y_{i},\;x_{i}\right);\;i=1,\;2,\ldots ,\;n\right\}$ into *k* subsets $D_{1},\;D_{2},\;{\ldots ,\;D}_{K}$ and let $D^{-k}=\;\left\{\left(y_{i},\;x_{i}\right)|\;\left(y_{i},\;x_{i}\right)\notin D_{k}\;\right\},\;k=1,\;2,\;\ldots ,\;K$. Then the estimate for β selection by *K*-fold cross-validation can be defined by:

(26) $$\begin{array}{l} D_{\beta_{0}}\left(\beta \right)=\;\frac{1}{K}\;\sum_{k=1}^{K}{L^{{\;}^{\prime }}}_{\beta_{0}}\left({\hat{\theta }}_{\beta }\backslash D_{k}\right) \end{array}$$

(27) $$\begin{array}{l} \text{where }{L_{\beta_{0}}}^{\prime }\left({\hat{\theta }}_{\beta }\backslash D_{k}\right)=\;\frac{1}{\beta }[1-\frac{1}{n_{k\;}{l_{\beta }}_{0}\left({\hat{\theta }}_{\beta }\right)}\sum_{y\in D_{k}}\\ \;{\left\{f\left(y\backslash {\hat{\theta }}_{\beta }\right)\right\}}^{\beta_{0}}] \end{array}$$

(28) $$\begin{array}{l} \text{with }l_{\beta_{0}^{\;}}\left({\hat{\theta }}_{\beta }\right)=\frac{1}{1+\beta_{0}}[\int {\left\{f\left(y|{\hat{\theta }}_{\beta }\right)\right\}}^{1+\beta_{0}}] \end{array}$$

Here ${\hat{\theta }}_{\beta }=\left({\hat{\gamma }}_{\beta },{\hat{\sigma }}_{\beta }^{2}\right)$ are estimated iteratively using Equations 16–17 based on the sub-dataset $D^{-k}$ and *n*_*k*_ is the number of observations in the sub-unit *D*_*k*_. We choose a suitable β by the minimizer of *D*_β_0__(β) for β. If the minimizer of *D*_β_0__(β) find β > 0, it indicates that the dataset is contaminated by outliers. If it shows β = 0, which indicates that the dataset is not contaminated by outliers and the proposed method reduces to the MLE based regression approach. See Mollah et al. (2007, 2010) for more discussion about β selection by cross-validation.

### Simulated Data

We have considered three different scenarios to investigate the performance of the proposed methods compared with most popular robust methods such as M-Huber, M-Hampel, M-Tukey, LMS, LTS, MM (Yohai, 1987), S (Rousseeuw and Yohai, 1984) and fast-S (Salibian-Barrera and Yohai, 2006) using simulation data. The details procedures for generating simulated data are given below:

**Scenario-1:** To investigate the influence of outlying observations in our proposed method in comparison with some popular existing methods as early mentioned, first we have generated a clean dataset that contains *n* = 100 pairs of observations (*y*_*i*_*, x*_1*i*_), *i* = 1, 2,…, 100 satisfying two-variable regression model with *p* = 1 in equation (1) by taking γ_0_ = 1.0, γ_1_ = 6.0 and assuming *u* ~ NID (0,σ^2^ = 0.5). Then we contaminated few observations in *y* (response/dependent variable) artificially multiplying by 3 to 5 folds from random position to obtain three contaminated datasets having outlying observations 15, 30, and 45% in *y*, respectively. We have replicated each case 100 times to generate 100 datasets for each case of outliers and applied all methods, including the proposed method, to estimate the regression parameters in each case of outliers. To investigate the performance, we computed mean square error (MSE) defined as $MSE\left({\hat{\gamma }}_{i}\right)=E_{\gamma }\left[{\left({\hat{\gamma }}_{i}-\gamma_{i}\right)}^{2}\right]=Var\left({\hat{\gamma }}_{i}\right)+{\left[E\left({\hat{\gamma }}_{i}\right)-\gamma_{i}\right]}^{2}=\overset{100}{\underset{j=1}{\sum }}{\left({\hat{\gamma }}_{ij}-\gamma_{i}\right)}^{2}/100$, where ${\hat{\gamma }}_{ij}\;$is the estimate of the regression parameter γ_*ij*_ in the *j*th replication and *i* = 0, 1. To investigate the performance with two or more explanatory variables, we have generated 100 observations for the response variable (*y*) as before by using the linear regression model (1) with the number of explanatory variables *p* = 1, 10, 20, 30, and 40 and assuming *u* ~ NID (0,σ^2^ = 0.5) in each case. Then we contaminated few observations in *y* (response/dependent variable) artificially multiplying by 3 to 5 folds from random position to obtain three contaminated datasets having outlying observations 15%, 30%, and 45% in *y*, respectively as before. We replicated each case 100 times as before to generate 100 datasets for each case and applied all methods, including the proposed method, to estimate the regression parameters in each case. Then MSE of the estimates have computed for the regression parameters $\gamma ={\left(\gamma_{0},\gamma_{1}{,\;\ldots ,\;\gamma }_{p}\right)}^{\prime }$ based on 100 replications. The average MSE of the estimates $\hat{\gamma }$ is calculated using $MSE_{p}\left(\hat{\gamma }\right)=\overset{100}{\underset{j=1}{\sum }}{\overset{p}{\underset{i=0}{\sum }}\left({\hat{\gamma }}_{ij}-\gamma_{i}\right)}^{2}/100\left(p+1\right)$, for *p* = 1, 10, 20, 30, and 40, respectively, where ${\hat{\gamma }}_{ij}$ is the estimates of γ_*i*_ in the *j*th replication.

**Scenario 2:** To investigate the influence of high leverage points (HLPs) in our proposed method in comparison some popular existing methods as early mentioned, first we have generated a clean dataset that contains *n* = 100 pairs of observations (*y*_*i*_*, x*_1*i*_), *i* = 1, 2,…, 100 satisfying two variables regression model with *p* = 1 in Equation (1) as before by taking γ_0_ = 1.0, γ_1_ = 6.0 and assuming *u* ~ NID (0, σ^2^ = 0.5). Then we contaminated few observations in *x*_1_ (independent variable) artificially multiplying by 3 to 5 folds from random position to obtain four contaminated datasets having HLPs 5, 15, 30, and 45% in *x*_1_, respectively. We have replicated each case 100 times to generate 100 datasets for each case of HLPs and applied all methods, including the proposed method, to estimate the regression parameters in each case of HLPs.

**Scenario 3:** Data were generated based on linear model (1) with *p* > 1 independent and identically distributed *N (0, 1)* predictors (*p* = 2, 8) and sample sizes *n* = 20, 50, 100, 200, 500, where all slopes and intercepts were set to 0 and error follows normal. In this case, 500 datasets were generated for each combination of *p, n*, and error distribution. The relative efficiency (RE) is calculated by using equation:

$$\begin{array}{l} RE\;=\;\frac{\sum_{j=1}^{500}{\left\|{\hat{\gamma }}_{j}^{MLE}\right\|}^{2}}{\sum_{j=1}^{500}{\left\|{\hat{\gamma }}_{j}\;\right\|}^{2}}, \end{array}$$

the ratio of total mean squared errors. It is noted that, ${\hat{\gamma }}_{j}^{MLE}$ is the distribution-specific maximum likelihood estimator (MLE) with the clean dataset and ${\hat{\gamma }}_{j}$ is the study estimator.

### Real Datasets

We have used three freely available real datasets and one rice SNPs dataset generated in our laboratory for association analysis to explore the performance of the proposed method in comparison of other methods:

- Dataset 1, education-related dataset studied by Coleman et al. (1966). The data set contained information on 20 schools from the Mid-Atlantic and New England states. Variables are described as *X*1: staff salaries per pupil, *X*2: percent of white-collar fathers, *X*3: socioeconomic status composite deviation, *X*4: mean teacher's verbal test score, *X*5: mean mother's educational level (one unit is equal to two school years), and *Y:* verbal mean test score (all sixth graders). This dataset was contaminated by HLPs. To investigate the performance of the algorithms, we re-contaminated this dataset by additional HLPs and outliers. The data set displayed in Supplementary Table S1. In Supplementary Table S1, *aa*(***bb***) means *aa* is replaced by ***bb*** for recontamination of the dataset.
- Dataset 2, mice hypertension data previously investigated by Sugiyama et al. (2001) which is available in the R/qtl package (Broman et al., 2003). This dataset was analyzed to investigate the genetic control of salt-induced hypertension in male mice obtained by backcrossing between the salt-sensitive c57BL/6J and the non-salt-sensitive A/J (A) inbred mouse strains.
- Dataset 3, iron status for liver and spleen dataset in mice studied by Grant et al. (2006). A total of 284 F_2_ mice were bred from F_1_ with either C57BL/6J/Ola or SWR/Ola strains, and this data are available in R/qtlbook package as the iron data. The iron level (in μg/g) in the liver and spleen are the two phenotypes, and the ratio of males and females are almost equal in this dataset (Grant et al., 2006). We have used log_10_ values for analysis of the data as the distribution was skewed from normality according to the previous study (Grant et al., 2006).
- Dataset 4, a total of 138 RILs (recombinant inbred lines) population obtained from Xieyou9308, super hybrid rice, was used for the association study of both rice quality traits chalkiness degree (CD) and chalkiness percentage (CP) in this study. The details about field experiments and genotypes of DNA resequencing are described in the materials and method section in our previous study (Xu et al., 2015). We have obtained 701,867 SNPs from DNA resequencing. Then we remove the low-frequency SNPs (minor allele frequency <5%) and pruning LD correlated SNPs (*r*^2^ > 0.4) using Plink. Subsequently, we have applied a generalized multifactor dimensionality reduction procedure to screen potential SNPs associated with traits (Zhu et al., 2013). Finally, we applied our proposed, LS, RLS, and fast-S methods to identify the important SNPs associated with CD and CP. The simple linear regression model was considered, where we included single SNPs in the model to estimate the effect of each SNPs, and the chi-square test was used for testing the significance of the SNPs.

### Annotation of SNPs and Genes

We have used CARMO (comprehensive annotation of rice multi-omics data) for SNP annotation (Wang et al., 2015). Candidate genes of the identified SNPs were derived from the RAP-DB (http://rapdb.dna.affrc.go.jp/) and Oryzabase (https://shigen.nig.ac.jp/rice/oryzabase/). Moreover, we used different webpages such as PubMed, Google Scholar, Uniport, and Web of Science to know the functions of the identified genes.

## Results

### Simulation Data Analysis Results

#### Performance Investigation in the Presence of Outliers Using Simulation Scenario 1

**Figure 1:**
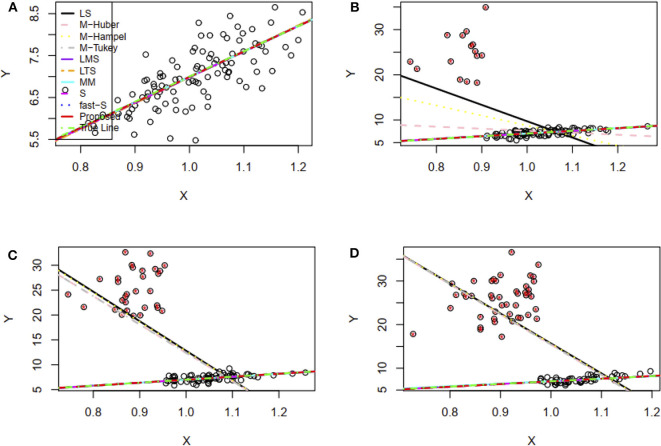
Simulation results of the two-variable regression model to fit regression lines. **(A–D)** The fitted regression line in absence and presence of 15, 30, and 45% outliers, respectively. LS, least-squares estimator; M-Huber, M-Huber estimator; M-Hampel, M-Hampel estimator; M-Tukey, M-Tukey estimator; LMS, least median of squares estimator; LTS, least trimmed squares estimator; MM, MM-estimator; S, S-estimator; fast-S, fast-S estimator; Proposed, Proposed estimator.

Figure 1A represents the fitted regression lines by all methods, as mentioned in the method section with our proposed method in the absence of outliers. The straight lines represented by a solid line with black color, dash-dot line with pink color, dot line with yellow color, dot-dash line with gray color, a solid line with purple color, dot-dash line with orange color, a solid line with cyan color, dash-dot line with magenta color, dot-dot line with blue color, the solid line with red color, and dot-dash line with green color for LS, M-Huber, M-Hampel, M-Tukey, LMS, LTS, MM, S, the fast-S, proposed method and True line, respectively. We observe that all methods including the proposed method produce reasonable line similar to the true line (dot-dash line with green color), where the true line has drawn using the actual parameter γ_0_ = 1.0 and γ_1_ = 6.0 (Figure 1A). Figures 1B–D shows the fitted regression line with these three contaminated datasets by all methods, including our proposed method, and the descriptions of lines in these figures are the same as Figure 1A. Results showed that the proposed method, as well as LMS, LTS, MM, S, and fast-S, produced straight lines similar to the real line, while the other methods fail to produce reasonable lines for all of the three contaminated datasets (Figures 1B–D).

**Figure 2:**
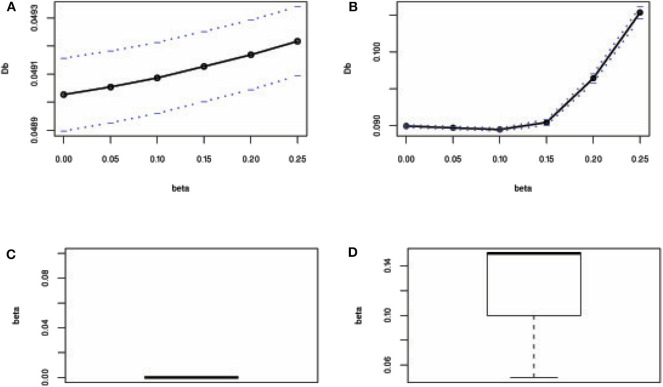
β-selection by cross-validation (CV) and the box plot of beta values. **(A,B)** Beta-selection results using simulation data in the absence and presence of outlier, respectively. **(C,D)** Box plot of beta values without and with outliers, respectively.

Besides, mean square error (MSE) has used to test the efficiency of the estimates and accordingly 100 datasets generated by replication for each case as mentioned above in this study. MSE results show that the proposed method's MSEs are smaller than other method's MSEs in most of the cases (Supplementary Table S2). An estimator having smaller MSE is said to be a good estimator. These results indicated that the proposed method's estimators are efficient estimators. The estimated values of the regression parameters and the MSE of the estimates are given in Supplementary Table S2. However, the performance of the proposed method depends on the value of the tuning parameter β. We observe that the proposed method produces reasonable estimates for any values of β within the range [0, 0.25] and [0.05, 0.25] for all uncontaminated and contaminated datasets in this setting, respectively (Figures 2A,B). Since smaller β decreases the power of robustness but increases the efficiency of the estimates and vice-versa for the larger β (Basu et al., 1998), we select β = 0.2 to control the trade-off between robustness and efficiency of the estimates for all datasets in this setting. Additionally, the box plot results show that the estimated values of β were nearby zero and >0.14 in the absence and presence of outliers, respectively (Figures 2C,D). Since the β-MLE estimators with β = 0 reduces to the classical MLE, the above results indicated that the proposed estimators reduce to the classical MLE in the absence of outliers.

**Figure 3:**
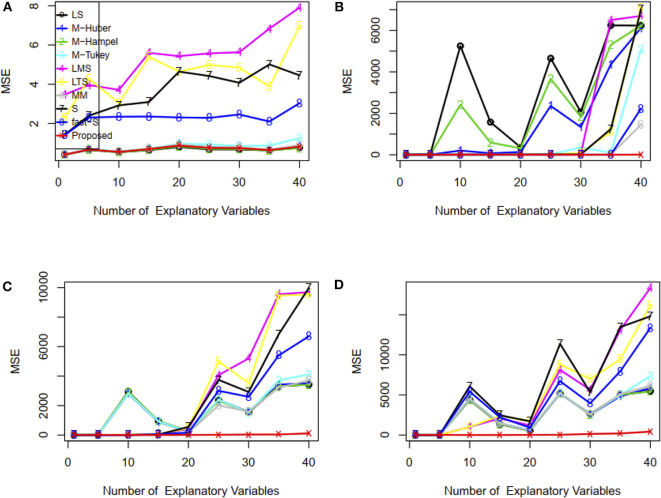
Plots of MSE with respect to the number of explanatory variables. **(A–D)** In the absence and presence of 15, 30, and 45% outliers, respectively. LS, least-squares estimator; M-Huber, M-Huber estimator; M-Hampel, M-Hampel estimator; M-Tukey, M-Tukey estimator; LMS, least median of squares estimator; LTS, least trimmed squares estimator; MM, MM-estimator; S, S-estimator; fast-S, fast-S estimator; Proposed, Proposed estimator.

Furthermore, we have investigated the performance of the proposed method when the number of explanatory variables is increased in the regression model. Results showed that LS, M-Tukey, and the proposed method produce smaller MSE than other methods for each case of explanatory variables in the absence of outliers (Figure 3A). In the case of 15% outliers, results show that only MM, fast-S, and the proposed method produced almost stable and smaller MSE than other methods in each case of explanatory variables increased in the model (Figure 3B). However, in the case of 30 and 45% outliers in the datasets, only the proposed method produces stable and smaller MSE than other methods in each case of explanatory variables (Figures 3C,D). MSE values for each case of explanatory variables are given in Supplementary Table S3. These results indicate that the proposed method shows better performance than traditional methods (M-Huber, M-Hampel, M-Tukey, LTS, LMS, MM, S, and fast-S) in case of both absence and presence of outliers.

#### Performance Investigation in the Presence of High Leverage Points (HLP) Using Simulation Scenario 2

**Figure 4:**
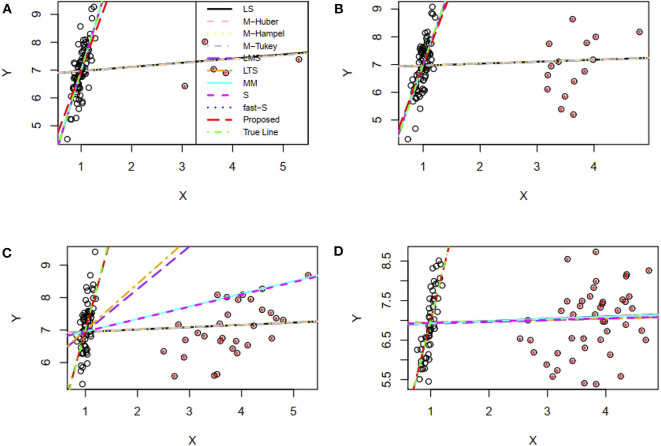
Simulation results for two-variable regression analyses in the presence of high leverage points. **(A–D)** The fitted regression line in the presence of 5, 15, 30, and 45% high leverage points, respectively. LS, least-squares estimator; M-Huber, M-Huber estimator; M-Hampel, M-Hampel estimator; M-Tukey, M-Tukey estimator; LMS, least median of squares estimator; LTS, least trimmed squares estimator; MM, MM-estimator; S, S-estimator; fast-S, fast-S estimator; Proposed, Proposed estimator.

In this study, the performance of the proposed method was investigated in the presence of 5, 15, 30, and 45% contaminated high leverage points (HLP) in the dataset. Then all of the methods, including the proposed method, were applied to fit the regression line for each case of contamination. Results showed that the proposed method including LMS, LTS, MM, S, and fast-S produced straight lines similar to the true line in the presence of 5 and 15% HLP in the dataset, while the other methods failed to produce reasonable lines in these cases, where the true line is drawn using the true values of the parameter γ_0_ = 1.0 and γ_1_ = 6.0 in the regression model (Figures 4A,B). Interestingly, results showed that only the proposed method produces reasonable lines similar to the true line, while all the other methods fail to produce reasonable lines in cases of 35 and 45% HLPs (Figures 4C,D). Similar to the previous section, estimator efficiency was also investigated by generating 100 datasets with replications for each case, as stated above. The average estimated values of the regression parameters and the MSE of the estimates are given in Supplementary Table S4. Results showed that the MSEs of the proposed estimates are smaller than other estimates in most of the cases, indicating that the proposed method performed better also in cases of HLPs in the datasets (Supplementary Table S4).

#### Performance Investigation Using Relative Efficiency in Case of Simulation Scenario 3

**Figure 5:**
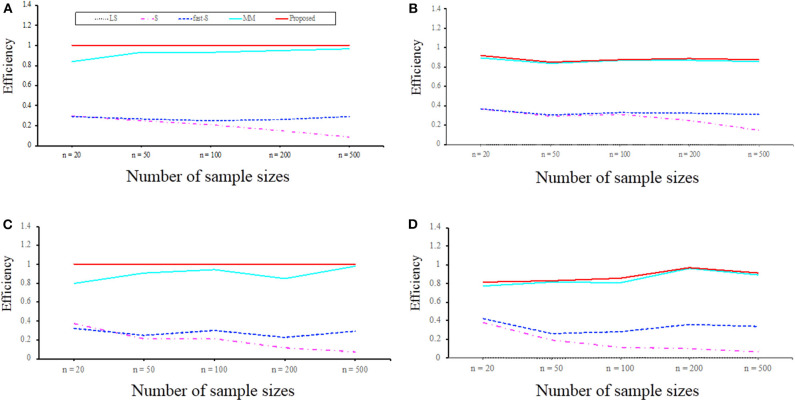
Efficiency with respect to maximum likelihood estimator (MLE) for *p* = 2 and *p* = 8 variables regression model with normal errors for sample sizes *n* = 20, 50, 100, 200, and 500. **(A,B)** Efficiency concerning MLE for *p* = 2 in the absence of outliers and the presence of 10% outliers, respectively. **(C,D)** Efficiency concerning MLE for *p* = 8 in the absence of outliers and the presence of 10% outliers, respectively. LS, least-squares; S, S-estimator; fast-S, fast-S estimator; MM, MM-estimator; Proposed, Proposed estimator.

To examine the performance of the proposed method in point of finite sample efficiency and robustness, the proposed method results were compared with the most well-known estimator S, MM, and fast-S. Details about data are given in the method and material section. Figure 5 displays efficiency results for linear regression with *p* = 2 and *p* = 8, respectively. The overall summary from the results is that the proposed estimator is highly efficient, even with sample sizes as small as *n* = 20 or *n* = 500. It is worth mentioning that the proposed estimator is asymptotically more efficient than S, MM, and fast-S estimators. We also investigate the efficiencies in case of data contamination, and results show that S, MM, and fast-S estimators less efficient than the proposed estimator (Figure 5). Therefore, we conclude that the efficiencies of the proposed estimator for normal error distribution are less affected by high dimension and outliers than the three popular estimators such as S, MM, and fast-S.

### Real Data Analysis Results

#### Performance Investigation Based on Coleman Data

**Figure 6:**
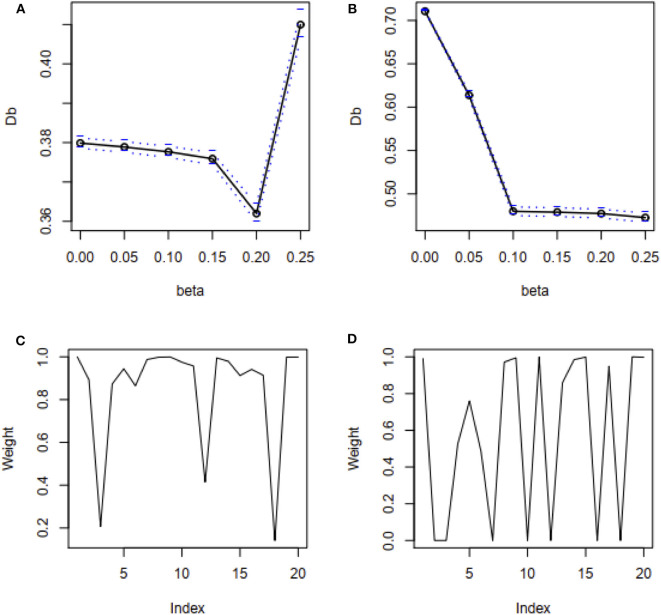
β-selection by cross-validation (CV) and β-weight plot. **(A,B)** β-selection by cross-validation (CV) for original and recontaminated datasets, respectively. **(C,D)** β-weight plot for original and recontaminated data, respectively.

We have used the real dataset drawn from a population studied by Coleman et al. (1966) to reveal the performance of the proposed method in comparison with LS, RLS, and fast-S method. Details information of this dataset is given in the method and material section. Mosteller and Tukey (1977) analyzed this dataset using LS and RLS (reweighted LS), and the results are also described by Rousseeuw and Leroy (1987). They reported that RLS showed better performance than LS. To compare their results with the fast-S and our proposed method, we applied both algorithms in the original dataset. Then we have applied LS, RLS, fast-S, and the proposed method on the contaminated dataset. To apply our proposed method, first we have selected the appropriate β by cross-validation for both datasets. Figures 6A,B shows the cross-validation results for original and contaminated datasets, respectively. Using “one standard error rule” we select β = 0.2 for both datasets. Then we applied our proposed method with β = 0.2 in both datasets. The proposed method produces β-weight for each data point of both datasets. Figures 6C,D shows the β-weights for each data point of original and contaminated datasets, respectively. From Figure 6C, we observe that data points 3, 13 and 19 are contaminated by outliers/HLPs in the original dataset, while the β-weight plot in Figure 6D indicates data points 3, 4, 7, 10, 12, 16, and 18 are contaminated by outliers/HLPs in the contaminated dataset. The results for both datasets are given in Table 1. We observe that the LS method shows only *X*3 and *X*4 are significant with *p* < 0.05 for the original dataset, while RLS, fast-S, and the proposed method showed all explanatory variables are significant for regression analysis with *p* < 0.05.

**Table 1:** Results for coleman data by LS, RLS, fast-S, and the proposed method.

**Methods**	**LS**		**RLS**		**fast–S**		**Proposed**	
**Variable**	**Coeff**.	***P*-value**	**Coeff**.	***P*-value**	**Coeff**.	***P-*value**	**Coeff**.	***P*-value**
**Results for the original Coleman dataset**								
x1	−1.79	0.17	−1.2	0.03	−1.64	0	−1.61	0
x2	0.04	0.43	0.08	0	0.08	0	0.09	0
x3	0.56	0	0.66	0	0.66	0	0.65	0
x4	1.11	0.02	1.09	0	1.31	0	1.31	0
x5	−1.81	0.39	−3.89	0	−4.15	0	−4.02	0
Constant	19.95	0.17	29.75	0	26.76	0	26.76	0
**Results for the recontaminated Coleman dataset**								
x1	−18.8	0.49	−17.31	0.54	−16.45	0.55	−2.02	0
x2	−0.78	0.19	−0.63	0.22	−0.97	0.01	0.06	0
x3	−0.98	0.27	−0.79	0.53	−1.32	0.01	0.64	0
x4	6.97	0.45	7.02	0.37	9.27	0.48	1.42	0
x5	32.85	0.16	35.47	0.32	48.07	0.29	−2.64	0
Constant	−236.38	0.31	−257.27	0.46	−397.19	0.28	16.55	0

*LS, least-squares; RLS, reweighted least-square; Coeff, Coefficient*.

It should be noted here that results for LS and RLS methods are given in Rousseeuw and Leroy (1987). However, in the case of the contaminated dataset, only the proposed method showed that all explanatory variables are significant for regression analysis with a *p* < 0.05. Thus, it could be concluded that the proposed method is more stable than LS, RLS, and fast-S methods for regression analysis.

#### Detection of Essential Genes (QTLs) Influencing Hypertension of Mice Real Dataset

**Figure 7:**
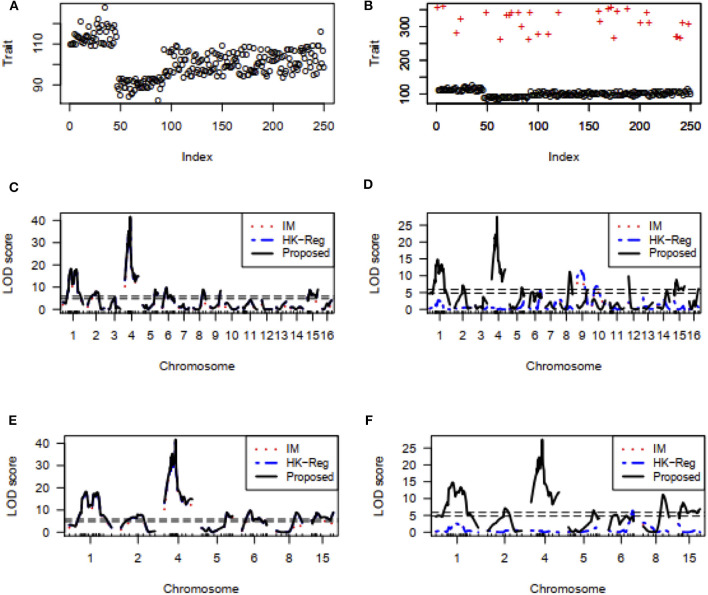
Mouse Genome data analysis results for the backcross population. **(A,B)** Scatter plot of real traits (hypertension of mice) with the indexes of their sampling units in the absence and presence of phenotypic outliers, respectively. **(C,D)** LOD scores by the IM, HK-Regression, and the proposed methods in the absence and presence of 10% outliers, respectively. **(E–F)** Specific chromosomes LOD scores by the IM, HK-Regression, and the proposed methods in the absence and presence of 10% outliers, respectively.

We also investigate the performance of our proposed method for detecting important genes influencing hypertension of mice in comparison of traditional interval mapping (IM) (Lander and Botstein, 1989) and HK-Reg (Haley and Knott, 1992) model using the real dataset of Sugiyama et al. (2001). Figure 7A represents the blood pressure (hypertension) of 250 male progeny backcrosses to B6. Figure 7B shows the scatter plot of the contaminated dataset with outliers (+). Figure 7C shows that two QTLs on chromosome 1 and two QTL on chromosome 4 are statistically highly significant, and one QTL on each of chromosomes 2, 5, 6, 8, and 15 are suggestive by all three methods for the uncontaminated real dataset. For more clearly observe the results, we draw Figures 7E,F, which showed the same result as in Figures 7C,D for the specific chromosomes. However, in the presence of outliers, almost similar results are obtained only by the proposed method, as shown in Figure 7D, which indicates that the proposed method significantly improves the performance over the traditional IM and HK- Regression methods.

#### Identification of Vital QTLs Regulating the Basal Liver and Spleen Iron Level in Mice

**Figure 8:**
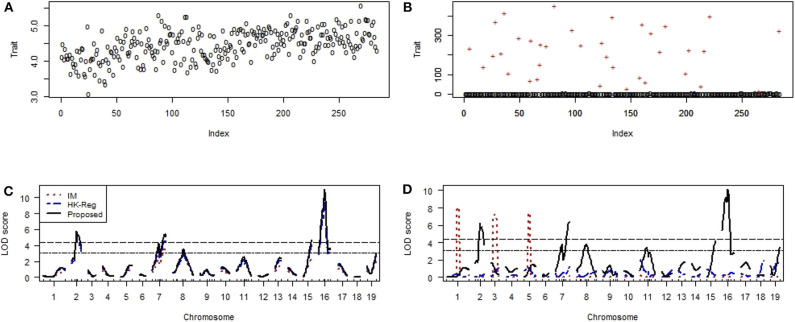
Genomic scan for the iron status in the liver of combined male and female sexes. **(A,B)** Scatter plot of real traits (iron level in the liver) with the indexes of their sampling units in the absence and presence of phenotypic outliers, respectively. **(C,D)** LOD scores by the IM, HK-Reg, and the proposed methods in the absence and presence of 10% outliers, respectively.

We have used another mice data set to investigate the performance of the proposed method to compare with the traditional methods (IM and HK-Reg) to identify the crucial genes (QTLs) controlling the iron status in mice. Scatter plot of the non-heme iron level in the liver of 284 mice in absence and contaminated dataset with 10% outliers (+) represent by Figures 8A,B, respectively. QTL search results show that two highly significant (*P* < 0.01) polymorphic loci on chromosomes 2 and 16 and four suggestive QTLs on chromosomes 8, 11, 15, and 19 were identified by interval mapping (IM), HK-Reg and proposed method when using the original data (Figure 8C). We have artificially contaminated this dataset by 10% outliers to investigate the performance of our proposed method compared with existing methods. Results show that IM gave misleading results and failed to detect QTL positions correctly in the presence of outliers (Figure 8D). HK-Reg method was also unable to detect the QTL position in the absence of outliers (Figure 8D). However, the proposed method gives almost similar results in the presence of outliers for the iron level in the liver (Figure 8D).

**Figure 9:**
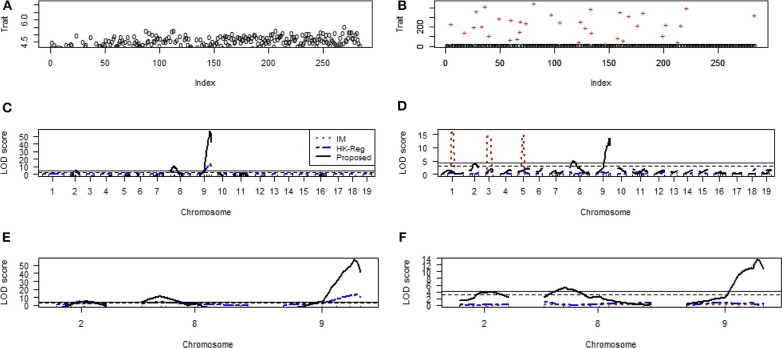
Genomic scan for the iron status in the spleen of combined male and female sexes. **(A,B)** Scatter plot of real traits (iron level in spleen) with the indexes of their sampling units in the absence and presence of phenotypic outliers, respectively. **(C,D)** LOD scores by the IM, HK-Reg, and the proposed methods in the absence and presence of 10% outliers, respectively. **(E,F)** Specific chromosomes LOD scores by the IM, HK-Reg, and the proposed methods in the absence and presence of 10% outliers, respectively.

Figures 9A,B shows the scatter plot of the non-heme iron level in the spleen of 284 mice in the absence and contaminated dataset with 10% outliers (+), respectively. We have applied the same procedure to generate the artificial dataset, as mention above. QTL analysis results show that one locus in each chromosome 8 and 9 was highly significant (*p* < 0.01) and one suggestive QTL on chromosomes 2 were identified by IM, HK-Reg and proposed method in the absence of outliers but only proposed method identified the original position of these QTLs in the presence of 10% outliers for spleen variable (Figures 9C,D). We have only considered the identified QTLs and their positions in Figures 9E,F to observe the results clearly demonstrated in Figures 9C,D. These results suggest that the proposed method considerably improves the performance over the traditional IM and HK- Regression methods for identifying the QTL determine the basal iron status in the liver and spleen. All of the real data (education, mice hypertension, and iron data) analysis results show that the proposed method demonstrates robust properties with respect to data contaminations, overcome the weakness of the traditional methods, and identify the vital gene influencing hypertension and iron level in the liver and spleen of mice.

#### Identification of Important SNPs Associated With Rice Quality Traits

Chalkiness is an essential factor influencing the grain character of rice and principally governed by genetic elements (Zhu et al., 2018). Chalkiness percentage (CP), the ratio of total grains that is chalky, and chalkiness degree (CD), the proportion of the whole area of a kernel, are two key characteristics of rice grain. We have compared the results of the proposed method with the most well-known least squares (LS), reweighted least square (RLS), and a robust method, namely fast-S, to investigate the performance. A total of 15 significant SNPs that were significantly associated with the chalkiness degree identified by our proposed method, whereas only 10 SNPs identified by both LS and RLS method with *p* < 1.4 × 10^−5^ (Table 2). However, only three significant SNP identified by the fast-S method for the CD variable (Table 3). The genomic position, SNP location, annotation, and gene symbol synonym of each SNP for CD variable is given in Supplementary Table S5. Results showed that five SNPs corresponded to genes, which are encoded a hypothetical protein. SNP rs34767210 lies in the intronic region of the candidate gene *OsRH3*, which is encoded similar to ATP-dependent RNA helicase. Another SNP rs257317 lies in the exonic region of the candidate gene *OsPGIP4*, which encodes polygalacturonase-inhibiting proteins. Furthermore, another SNP rs24313516 lies in the upstream part of the candidate gene *OsMOC1* (*Os06g0610300*), which encodes hypothetical protein.

**Table 2:** SNPs identification for chalkiness degree (CD) of rice controlling by only additive effects using LS, RLS, fast-S, and the proposed method.

**Identified SNPs**	**Chr**.	**Physical position**	**Allele**	**Methods**							
				**LS**		**RLS**		**fast-S**		**Proposed**	
				**Estimate**	***P*-value**	**Estimate**	***P*-value**	**Estimate**	***P*-value**	**Estimate**	***P*-value**
rs26502245	2	26502245	C/T	6.16	1.5E-07	5.67	1.0E-07	**5.23**	**6.6E-05**	6.16	1.1E-07
rs26788061	2	26788061	C/T	**5.15**	**1.9E-05**	5.19	8.8E-07	5.22	5.4E-06	5.16	1.3E-05
rs26937350	2	26937350	C/A	**5.16**	**1.7E-05**	4.70	7.0E-06	**4.40**	**2.2E-04**	5.15	1.4E-05
rs29639996	2	29639996	T/C	**5.16**	**1.7E-05**	4.33	3.1E-05	**3.96**	**2.8E-03**	5.14	1.4E-05
rs34767210	3	34767210	A/G	5.18	1.4E-05	**4.45**	**1.9E-05**	**4.18**	**1.4E-03**	5.17	1.1E-05
rs257317	5	257317	C/T	**5.18**	**1.5E-05**	**4.47**	**1.8E-05**	**4.25**	**2.3E-03**	5.18	1.2E-05
rs490822	5	490822	C/T	5.40	8.1E-06	4.61	1.0E-05	4.37	4.8E-03	5.39	6.8E-06
rs22257511	6	22257511	A/T	5.55	3.6E-06	4.81	4.5E-06	**4.49**	**3.5E-03**	5.54	3.0E-06
rs23935378	6	23935378	C/G	5.40	6.1E-06	4.73	6.1E-06	**4.01**	**1.2E-02**	5.39	4.9E-06
rs24313516	6	24313516	T/A	5.48	3.7E-06	4.57	1.2E-05	**4.30**	**2.8E-03**	5.47	3.1E-06
rs26192911	6	26192911	G/A	**5.18**	**1.5E-05**	**4.49**	**1.7E-05**	**4.28**	**4.8E-04**	5.17	1.2E-05
rs26894569	6	26894569	T/C	5.25	1.4E-05	5.07	1.5E-06	**4.86**	**2.7E-04**	5.25	1.1E-05
rs22686933	9	22686933	T/G	5.36	9.E-05	5.11	1.2E-06	4.88	7.9E-04	5.36	7.4E-06
rs1745509	11	1745509	G/T	5.46	6.7E-06	**4.40**	**2.4E-05**	**3.85**	**4.5E-02**	5.44	6.0E-06
rs16826302	11	16826302	T/C	5.36	7.2E-06	**4.34**	**3.0E-05**	**3.92**	**1.9E-02**	5.34	6.3E-06

*Chr, chromosome; LS, least-squares; RLS, reweighted least squares. We have considered Bonferroni-correction for identification of important SNP (P-value < 1.4E-05). The bolded black color indicated that these SNPs are not significant by the respective methods*.

**Table 3:** SNPs identification for chalkiness percentage (CP) of rice controlling by only additive effects using LS, RLS, fast-S, and the proposed method.

**Identified SNPs**	**Chr**.	**Physical position**	**Allele**	**Methods**							
				**LS**		**RLS**		**fast-S**		**Proposed**	
				**Estimate**	***P*-value**	**Estimate**	***P*-value**	**Estimate**	***P*-value**	**Estimate**	***P*-value**
rs26502245	2	26502245	C/T	18.93	1.8E-06	20.13	3.6E-39	19.89	6.6E-07	18.96	1.3E-06
rs35426658	2	35426658	C/T	19.12	1.5E-06	20.03	5.5E-39	19.43	5.3E-06	19.13	1.1E-06
rs4803005	1	4803005	A/T	19.68	1.2E-06	20.59	4.8E-40	19.94	3.7E-06	19.69	9.0E-07
rs28088550	4	28088550	C/T	17.92	9.9E-06	20.30	1.7E-39	19.54	2.1E-06	17.96	7.3E-06
rs154937	5	154937	G/A	18.00	1.2E-05	18.76	1.7E-36	**17.99**	**5.4E-05**	18.00	9.6E-06
rs490822	5	490822	C/T	18.93	4.1E-06	20.07	4.8E-39	**19.26**	**1.7E-05**	18.94	3.2E-06
rs646158	5	646158	G/A	17.38	1.6E-05	18.75	1.7E-36	**18.02**	**1.9E-05**	17.40	1.2E-05
rs653823	5	653823	G/A	18.49	4.4E-06	18.94	7.4E-37	**18.40**	**1.6E-05**	18.49	3.5E-06
rs753549	5	753549	G/T	17.48	1.1E-05	18.29	1.5E-35	17.86	1.5E-05	17.49	9.1E-06
rs22670304	7	22670304	T/A	−17.20	1.6E-05	−18.50	5.7E-36	−18.18	5.8E-06	−17.22	1.2E-05
rs25558512	7	25558512	T/C	−17.80	8.0E-06	−19.11	3.5E-37	−18.87	8.2E-06	−17.82	6.1E-06
rs25982235	7	25982235	G/A	−17.62	1.5E-05	−18.04	4.7E-35	**−17.52**	**3.1E-05**	−17.62	1.3E-05
rs26127356	7	26127356	G/A	−18.58	2.7E-06	−19.27	1.7E-37	−19.06	2.9E-06	−18.59	2.1E-06
rs26290041	7	26290041	A/G	−18.68	2.6E-06	−19.06	4.2E-37	−18.91	4.9E-06	−18.69	2.0E-06
rs26314656	7	26314656	G/A	−18.34	4.2E-06	−18.69	2.3E-36	**−18.09**	**1.9E-05**	−18.33	3.4E-06
rs26357917	7	26357917	G/A	**−17.27**	**1.7E-05**	−17.25	1.9E-33	**−16.94**	**6.9E-05**	−17.26	1.4E-05
rs26752914	7	26752914	G/A	−18.88	2.1E-06	−19.15	2.8E-37	−18.71	6.3E-06	−18.88	1.7E-06
rs26859579	7	26859579	G/A	−17.80	7.7E-06	−18.76	1.7E-36	−18.49	9.0E-06	−17.82	6.0E-06
rs14945421	9	14945421	T/C	17.81	9.3E-06	18.37	1.0E-35	18.43	6.1E-06	17.83	7.2E-06
rs16790346	9	16790346	G/A	17.69	1.1E-05	18.61	3.3E-36	18.33	4.6E-06	17.71	8.7E-06
rs17097438	9	17097438	A/G	17.28	1.5E-05	17.93	7.8E-35	**17.31**	**3.7E-05**	17.28	1.2E-05

*Chr, chromosome; LS, least squares, RLS, reweighted least squares. We have considered Bonferroni-correction for identification of important SNP (P-value < 1.62E-05). The bolded black color indicated that these SNPs are not significant by the respective methods*.

For the CP variable, a total of 21 significant SNPs identified by our proposed method and RLS method, whereas the LS method identified 20 SNPs, but the fast-S method identified only 13 SNPs with *P* < 1.6 × 10^−5^ in this study (Table 3). Identified SNPs and their related information are given in Supplementary Table S6. The identified SNPs were located in different regions of different genes. Among those SNPs, five SNPs lie in the intergenic region, which was encoded hypothetical protein (Supplementary Table S6). More importantly, six SNPs were corresponding to the specific genes and lay in upstream, downstream, intronic and intergenic regions, and other SNPs were involved in different functions (Supplementary Table S6). For example, SNP rs154937 lies in the upstream region corresponding to the variant of *Os05g0102600*, which is encoded zinc finger (Znf) containing protein. SNPs rs26357917 is the variant of genes *Os07g0634900* encoded MYB transcription factors (TFs) family, which is, lies in the downstream region. Another SNP rs25982235 lies in the intronic region of the corresponding gene *OsDEP1* (*Os07g0627000*), which is encoded chloroplastic alpha-glucanotransferase involved in maltotriose metabolism (https://www.uniprot.org/uniprot/Q8LI30). SNP rs16790346 is a variant of the *PERSISTENT TAPETAL CELL 1* gene that lies in the intergenic region. SNP rs25558512 lies in the intergenic region of the candidate gene *Os07g0618800*, which encodes a calmodulin-like protein. Also, SNP rs26127356 lies in the upstream of the *OsRNS8* (*Os07g0629900*), which encode Ribonuclease (RNases) T2 domain-containing protein. Two SNPs rs26502245 and rs490822 lie in the exonic and intergenic region of corresponding genes *Os02g0656100* and *Os05g0108450*, respectively, were common in both CD and CP variables. The above results indicated that several mechanisms might be involved for controlling rice grain quality, including Znf protein and transcription factors.

## Discussion

This paper proposed a new robust regression analysis approach by maximizing the β-likelihood function. The β-likelihood function is a limiting case of the log-likelihood function, and it is induced from β-divergence. The value of the important parameter β plays a critical role in the performance of the proposed method. An appropriate value for the tuning parameter β is selected by CV. The CV procedure finds β = 0 in the absence of outliers, while in the presence of outlying observations, it produces β > 0. The robustness properties of the proposed method are discussed using the influence function. Simulation and real data analysis were used to evaluate the performance of proposed β-estimators. Simulation results exhibit that the proposed method executes better than popular robust methods (i.e., M-Huber, M-Hampel, M-Tukey, LTS, LMS, MM, S, and fast-S) in case of both outliers and high leverage points. MSE of the proposed method is smaller than other methods for the small and large number of explanatory variables indicated that the proposed method's estimators are efficient estimators. Because it is said that an estimator having smaller MSE is a good estimator than others. Relative efficiency analysis results show that the proposed method outperforms than other robust methods (S, MM, and fast-S) in case of high dimension and outliers.

We performed GWAS on the different types of genome datasets for checking the performance of the proposed method to identify the biomarker genes/SNPs. Genome (mice hypertension and iron data) and education data analysis results demonstrated that the proposed method shows robust properties with respect data contaminations, overcome the drawback of the traditional methods and identify the essential variables related to education and vital gene influencing hypertension of mice and iron level in liver and spleen. Moreover, we introduced a new approach for GWAS to identify essential SNPs responsible for important agronomic traits. Grain quality consists of different sophisticated features such as appearance character, milled feature, nutritional characters, and cooking attribute, where grain appearance is correctly connected by grain shape and chalkiness (Zhu et al., 2018). High CP and CD are leading barriers to develop rice grain characteristics in China (Feng et al., 2017). Many QTLs have been identified, but only several rice chalkiness responsible QTLs were cloned or fine mapped due to the genetics difficulty and volatility of chalky quality till now (Zhu et al., 2018).

GWAS demonstrated that identified SNPs have different functions, including chalkiness specific function in this study. For example, SNPs rs34767210, rs257317, and rs24313516 corresponding to genes *OsRH3, OsPGIP4*, and *MOC1* (*MONOCULM 1*), respectively for CP. Research showed that different essential functions, including ribosome biogenesis, translation origination, RNA synthesis, alteration, segmentation, and degradation governed by DEAD-box RNA helicases (Linder and Jankowsky, 2011). PGIPs (Polygalacturonase-inhibiting proteins), which are usually leucine-rice repeat (LRR) proteins, are assumed to perform vital roles for improving the resistance against a bacterial pathogen and also protection rice vs. fungi (Feng et al., 2016). *MOC1*, first identified gene controlling tiller number, regulated the origination and extension of auxiliary meristems at both vegetative and generative periods (Xu et al., 2012). Consequently, tiller and panicle branches affected by the loss of function *MOC1* in rice (Xu et al., 2012).

We have identified six important SNPs associated with the chalkiness percentage in this study. Those SNPs were corresponding to different genes encoding Znf protein, TFs, calmodulin-like protein, and RNases T2 domain-containing protein. Research showed that Znf proteins have diverse functions, including gene transcription, translation, cell adhesion, protein folding, RNA packaging, chromatin remodeling, zinc identifying and lipid binding, and many more (Laity et al., 2001). A study showed that MYB protein regulating the hormonal reactions throughout seed sprouting and development (Ambawat et al., 2013). A recent study showed that the PTC1 gene regulating the tapetal growth and pollen formation in rice (Li et al., 2011). A target gene's expression could be activated or suppressed governing by plant calmodulin-like proteins as it binds DNA straightaway and functioning as TF (Hoeflich and Ikura, 2002). Another recent study demonstrated that *OsRNS8* belongs to the S-RNases subfamilies whose genes related to tissue and stress (MacIntosh et al., 2010).

In this study, we have proposed an MLE-based robust regression method by maximizing the β-likelihood function. The selection of the tuning parameter β plays a crucial role in the performance of the proposed method. Note that the proposed method reduces to the classical MLE-based regression analysis for β = 0 in case of absence of outliers in the data, and the value of the tuning parameter β will be higher than zero in the presence of outliers in the data, which will be selected by the cross-validation procedures. Thus, a suitable value of β might assure the trade-off between the robustness and efficiency of estimators. It might be recognized that the standard linear regression procedure would be more beneficial than any other regression method in case of the absence of the outliers in the data, but any robust regression methods would be better if there are any outliers in the datasets. More importantly, it is very demanding and time consuming for the researchers to use an appropriate regression method if datasets have outliers. In this context, our proposed robust method would be appropriate than any other regression methods.

The efficiency of an estimator depends on the MSE value, and an estimator having smaller MSE than other estimators is said to be a good estimator. Simulation results suggested that the proposed method gives efficient estimators than other methods mentioned here in case of outliers and HLPs, elsewhere it shows similar performance in comparison with traditional methods. Also, our real data analysis including education, mice hypertension, and iron data showed that the proposed method gives efficient estimators and demonstrates robust properties with respect data contaminations, overcome the weakness of the traditional methods, and identify the vital gene influencing hypertension and iron level in liver and spleen of mice. Using the rice SNPs data, we have identified some highly significant SNPs associated with grain chalkiness traits in rice in this study. The identified SNPs might be useful for rice breeders to characterize the genes responsible for grain quality traits. Further validation of these SNPs might confirm their functions and provide the new resources for grain quality-related genes in rice. These results showed that the performance of the proposed method was better than other methods in both the absence and presence of outliers for artificial and real data analysis. Taken together, we conclude that the proposed method offers an important basis for further understanding of the robust regression analysis, which might be applied in various fields, including business, genetics, and bioinformatics.

## Data Availability Statement

The plant SNP data generated for this article can be found here: https://figshare.com/s/1a3e7a233820d05fcdfe publicly available datasets were also used in this study. Information about these can be found in the article.

## Author Contributions

MM conceptualization of the proposed method. MA and MM developed the method. HX provided the SNPs data, managed and organized the project. MA and MS performed the simulation and real data analysis. MA wrote the manuscript. MM and HX critically revised this manuscript. All authors have read and approved the final manuscript.

## Conflict of Interest

The authors declare that the research was conducted in the absence of any commercial or financial relationships that could be construed as a potential conflict of interest.
