# Supplementary material for: Robustification of Linear Regression and Its Application in Genome-Wide Association Studies
Source: Front Genet. 2020 Jun 8;11:549. doi: 10.3389/fgene.2020.00549 (PMC7295010; doi:10.3389/fgene.2020.00549)
Supplement: Supplementary file 1 [file Data_Sheet_1.docx]

Supplementary Material

**Supplementary Tables**

**Supplementary Table S1 |** Coleman data, containing information on 20 schools from the mid-Atlantic and New England states.

| ***Index*** | **X1** | **X2** | **X3** | **X4** | | **X5** | | **Y** |
| --- | --- | --- | --- | --- | --- | --- | --- | --- |
| 1 | 3.83 | 28.87 | 7.20 | | 26.60 | | 6.19 | 37.01 |
| 2 | 2.89 | 20.1 | -11.71 | | 24.40 | | 5.17 | 26.51 |
| 3 | 2.86 | 69.05(**9.05**) | 12.32 | | 25.70 | | 7.04 | 36.51(**109**) |
| 4 | 2.92 | 65.4 | 14.28 | | 25.70 | | 7.10 | 40.70 |
| 5 | 3.06 | 29.59 | 6.31 | | 25.40 | | 6.15 | 37.10 |
| 6 | 2.07 | 44.82 | 6.16 | | 21.60 | | 6.41 | 33.90 |
| 7 | 2.52 | 77.37 | 12.70(**6**) | | 24.90 | | 6.86 | 41.80(**125**) |
| 8 | 2.45 | 24.67 | -0.17 | | 25.01 | | 5.78 | 33.40 |
| 9 | 3.13 | 65.01 | 9.85 | | 26.60 | | 6.51 | 41.01 |
| 10 | 2.44 | 9.99(2) | -0.05 | | 28.01 | | 5.57 | 37.20(**137**) |
| 11 | 2.09 | 12.20 | -12.86 | | 23.51 | | 5.62 | 23.30 |
| 12 | 2.52 | 22.55(**9**) | 0.92 | | 23.60 | | 5.34 | 35.20(**126**) |
| 13 | 2.22 | 14.30 | 4.77 | | 24.51 | | 5.80 | 34.90 |
| 14 | 2.67 | 31.79 | -0.96 | | 25.80 | | 6.19 | 33.10 |
| 15 | 2.71 | 11.61 | -16.04 | | 25.20 | | 5.62 | 22.70 |
| 16 | 3.14 | 68.47(**4**) | 10.62(**5**) | | 25.01 | | 6.94 | 39.70(**130**) |
| 17 | 3.54 | 42.64 | 2.66 | | 25.01 | | 6.33 | 31.80 |
| 18 | 2.52 | 16.70(**5**) | -10.99(**-51**) | | 24.80 | | 6.01 | 31.70(**155**) |
| 19 | 2.68 | 86.27 | 15.03 | | 25.51 | | 7.51 | 43.10 |
| 20 | 2.37 | 76.73 | 12.77 | | 24.51 | | 6.96 | 41.01 |

**Supplementary Table S2 |** Average estimates of the regression parameters for two variable regression in presence of 0%, 15%, 30%, and 45% outliers respectively for 100 replications.

| **Methods** | **Percentage of outliers in Y-axis** | | | | | | | |
| --- | --- | --- | --- | --- | --- | --- | --- | --- |
|  | 0% | | 15% | | 30% | | 45% | |
|  | $\gamma_{0}$=1 | $\gamma_{1}$=6 | $\gamma_{0}$=1 | $\gamma_{1}$=6 | $\gamma_{0}$=1 | $\gamma_{1}$=6 | $\gamma_{0}$=1 | $\gamma_{1}$=6 |
| LS | 1.0  (0.3) | 6.0  (0.3) | 46.4  (2086.1) | -36.7 (1839.0) | 71.6  (5014.1) | -58.9  (4249.8) | 84.7  (7089.7) | -68.9  (5687.9) |
| M-Huber | 1.0  (0.3) | 6.0  (0.3) | 11.8  (149.6) | -4.2  (133.4) | 68.8  (4624.4) | -56.6  (3942.6) | 85.2  (7167. 8) | -69.6  (5798.8) |
| M-Hampel | 1.0  (0.3) | 6.0  (0.3) | 32.4  (1248.7) | -23.6  (1105.1) | 71.1  (4942.9) | -58.5  (4193.8) | 84.9  (7125.7) | -69.2  (5725.2) |
| M-Tukey | 1.0  (0.3) | 6.0  (0.3) | 1.017  (0.72) | 5.9  (0.7) | 68.9  (4644.3) | -56.7  (3961.3) | 85.5  (7216.3) | -69.9  (5842.4) |
| LMS | 1.3  (2.1) | 5.7  (2.0) | 1.1  (2.4) | 5.9  (2.3) | 1.2  (4.2) | 5.8  (3.8) | 1.3  (4.7) | 5.7  (4.1) |
| LTS | 1.2  (2.0) | 5.8  (2.0) | 1.1  (2.7) | 5.9  (2.5) | 1.2  (3.1) | 5.8  (2.9) | 1.2  (2.1) | 5.9  (1.8) |
| MM | 1.0  (0.3) | 6.0  (0.3) | 1.0  (0.7) | 6.  (0.7) | 1.0  (1.2) | 6.0  (1.0) | 1.9  (24.6) | 5.2  (20.5) |
| S | 1.2  (1.1) | 5.8  (1.0) | 1.0  (1.3) | 6.0  (1.2) | 1.1  (1.6) | 5.9  (1.4) | 1.1  (1.8) | 6.0  (1.6) |
| fast-S | 1.2  (1.1) | 5.8  (1.1) | 60  (1.2) | 6.0  (1.1) | 1.1  (1.5) | 6.0  (1.3) | 1.0  (1.5) | 6.0  (1.3) |
| Proposed | 1.0  (0.3) | 6.0  (0.3) | 1.0  (0.8) | 6.0  (0.7) | 1.0  (1.2) | 6.0  (1.1) | 1.1  (1.6) | 5.9  (1.4) |
| 1. Note that the true values of the parameters are $\gamma_{0}$=1 and $\gamma_{1}$=6  2. Results in parenthesis indicates MSE of the estimates | | | | | | | | |

**Supplementary Table S3** **|** Average MSE for the estimates of the regression parameters with respect to number of explanatory variable with 100 replications.

| Number of explanatory variables (without contaminated response) | | | | | |
| --- | --- | --- | --- | --- | --- |
| Methods | **1** | **10** | **20** | **30** | **40** |
| LS | 0.40 | 0.54 | 0.79 | 0.67 | 0.77 |
| M-Huber | 0.41 | 0.56 | 0.90 | 0.73 | 0.81 |
| M-Hampel | 0.40 | 0.53 | 0.83 | 0.70 | 0.74 |
| M-Tukey | 0.41 | 0.56 | 0.96 | 0.84 | 1.24 |
| LMS | 3.47 | 3.71 | 5.43 | 5.63 | 7.91 |
| LTS | 2.28 | 3.04 | 4.65 | 4.85 | 6.95 |
| MM | 0.41 | 0.56 | 0.93 | 0.81 | 0.90 |
| S | 1.42 | 2.92 | 4.64 | 4.07 | 4.45 |
| fast-S | 1.40 | 2.34 | 2.31 | 2.46 | 3.03 |
| Proposed | 0.41 | 0.55 | 0.87 | 0.74 | 0.81 |
| Number of explanatory variables (with 15% contaminated response) | | | | | |
| Methods | **1** | **10** | **20** | **30** | **40** |
| LS | 4.39 | 1308.98 | 91.58 | 516.62 | 1567.78 |
| M-Huber | 2.61 | 81.22 | 37.28 | 331.98 | 1539.32 |
| M-Hampel | 3.36 | 666.68 | 80.25 | 458.46 | 1552.21 |
| M-Tukey | 2.14 | 1.40 | 0.96 | 77.70 | 1256.84 |
| LMS | 3.30 | 5.51 | 7.69 | 127.81 | 3310.44 |
| LTS | 2.95 | 5.25 | 9.46 | 41.27 | 3210.68 |
| MM | 1.99 | 1.37 | 0.97 | 0.99 | 376.75 |
| S | 1.22 | 5.28 | 7.19 | 15.28 | 3092.67 |
| fast-S | 1.19 | 2.26 | 2.24 | 2.02 | 576.63 |
| Proposed | 2.54 | 1.39 | 1.06 | 1.02 | 10.84 |
| Number of explanatory variables (with 30% contaminated response) | | | | | |
| Methods | **1** | **10** | **20** | **30** | **40** |
| LS | 14.20 | 2943.25 | 269.37 | 1536.076 | 3424.67 |
| M-Huber | 11.47 | 2812.29 | 250.79 | 1565.40 | 3519.17 |
| M-Hampel | 13.78 | 2924.50 | 267.40 | 1556.24 | 3413.81 |
| M-Tukey | 11.03 | 2821.66 | 255.85 | 1525.70 | 4108.88 |
| LMS | 2.95 | 12.04 | 429.68 | 5200.75 | 9694.75 |
| LTS | 2.56 | 11.76 | 472.91 | 3524.99 | 9532.75 |
| MM | 7.56 | 2.90 | 150.70 | 1593.87 | 3705.28 |
| S | 2.12 | 10.15 | 554.03 | 2921.35 | 9986.42 |
| fast-S | 2.03 | 3.39 | 169.23 | 2594.01 | 6698.47 |
| Proposed | 12.23 | 3.03 | 5.68 | 34.43 | 113.39 |
| Number of explanatory variables (with 45% contaminated response) | | | | | |
| Methods | **1** | **10** | **20** | **30** | **40** |
| LS | 21.67 | 4377.15 | 526.12 | 2630.10 | 5484.89 |
| M-Huber | 22.45 | 4498.73 | 573.58 | 2542.78 | 5856.36 |
| M-Hampel | 21.82 | 4403.38 | 533.78 | 2552.55 | 5418.69 |
| M-Tukey | 23.02 | 4503.83 | 578.22 | 2577.39 | 7355.32 |
| LMS | 3.76 | 1024.46 | 1200.66 | 5643.70 | 18354.55 |
| LTS | 2.73 | 1019.67 | 1744.29 | 6983.58 | 16192.05 |
| MM | 23.72 | 4494.70 | 571.74 | 2554.25 | 6146.60 |
| S | 3.89 | 6077.41 | 1714.37 | 5342.24 | 14810.13 |
| fast-S | 3.79 | 5413.12 | 914.14 | 3954.73 | 13366.74 |
| Proposed | 3.29 | 4.47 | 15.97 | 135.68 | 422.82 |

**Supplementary Table S4 |** Average estimates of the regression parameters for two variable regression in presence of 0%, 15%, 30% and 45% high-leverage points (HLPs), respectively.

| Methods | Percentage of outliers in X-axis (% of High -Leverage Points) | | | | | | | |
| --- | --- | --- | --- | --- | --- | --- | --- | --- |
|  | 5% | | 15% | | 30% | | 45% | |
|  | $\gamma_{0}$=1 | $\gamma_{1}$=6 | $\gamma_{0}$=1 | $\gamma_{1}$=6 | $\gamma_{0}$=1 | $\gamma_{1}$=6 | $\gamma_{0}$=1 | $\gamma_{1}$=6 |
| LS | 6.8  (34.1) | 0.2  (34.2) | 6.0  (34.8) | 0. 1  (35.2) | 6.9  (34.5) | 0.1  (35.2) | 6.9  (34.6) | 0.1  (35.3) |
| M-Huber | 6.8  (34.2) | 0.2  (34.2) | 6.9  (34.8) | 0.1  (35.2) | 6.9  (34.5) | 0.1  (35.2) | 6.9  (34.6) | 0.1  (35.3) |
| M-Hampel | 6.8  (34.2) | 0.2  (34.2) | 6.9  (34.8) | 0.1  (35.2) | 6.9  (34.5) | 0.1  (35.2) | 6.9  (34.6) | 0.1  (35.3) |
| M-Tukey | 6.8  (34.2) | 0.2  (34.2) | 6.9  (34.8) | 0.2  (35.2) | 6.9  (34.5) | 0.1  (35.2) | 6.9  (34.6) | 0.1  (35.3) |
| LMS | 0.7  (1.8) | 6.3  (1.8) | 1.3  (3.4) | 5.7  (3.4) | 5.4  (27.4) | 1.0  (27.8) | 6.9  (35.2) | 0.0  (35.5) |
| LTS | 0. 7  (2.2) | 6.3  (2. 2) | 1.1  (3.9) | 5.9  (3.9) | 5.0  (25.8) | 1.9  (25.8) | 6.9  (35.2) | 0.0  (35.5) |
| MM | 1.0  (0.3) | 6.0  (0.3) | 1.3  (2.4) | 5.7  (2.5) | 6.6  (33.1) | 0.3  (33.8) | 6.9  (34.6) | 0.1  (35.3) |
| S | 0.8  (0.9) | 6.2  (0.9) | 1.2  (3.1) | 5.7  (3.2) | 6.6  (33.4) | 0.3  (33.9) | 6.9  (35.3) | 0.0  (35.6) |
| fast-S | 0.8  (0.9) | 6.2  (0.8) | 1.3  (3.2) | 5.7  (3.2) | 6.6  (33.4) | 0.3  (33.6) | 6.4  (35.3) | 0.0  (35.6) |
| Proposed | 1.1  (0.8) | 5.9  (0.9) | 1.1  (0.9) | 5.9  (1.1) | 1.2  (1.8) | 5.8  (1.9) | 1.0  (0.9) | 6.6  (0.9) |
| 1. Note that the true values of the parameters are $\gamma_{0}$=1 and $\gamma_{1}$=6  2. Results in parenthesis indicates MSE of the estimates | | | | | | | | |

**Supplementary Table S5 |** List of identified SNP and associated candidate gene regulating chalkiness degree (CD) in rice.

| SNP | Candidate Genes | SNP location^a^ | Annotation^b^ | Gene Symbol Synonym^c^ |
| --- | --- | --- | --- | --- |
| rs26502245 | Os02g0656100 | exonic | Conserved hypothetical protein. |  |
| rs26788061 | Os02g0661500 | downstream | Non-protein coding transcript. |  |
| rs26937350 | Os02g0664150 | UTR3 | Hypothetical gene. |  |
| rs29639996 | Os02g0714700 | downstream | Hypothetical protein. |  |
| rs34767210 | Os03g0827700 | intronic | Similar to ATP-dependent RNA helicase | *OsRH3* |
| rs490822 | Os05g0108450 | intergenic | Hypothetical gene. |  |
| rs257317 | Os05g0104700 | exonic | Leucine-rich repeat (LRR) protein, Inhibitor of fungal polygalacturonase, Defence response | *OsPGIP4* |
| rs22257511 | Os06g0573900 | downstream | Similar to 1-aminocyclopropane-1-carboxylic acid oxidase. |  |
| rs24313516 | Os06g0610300 | upstream | Conserved hypothetical protein. | *OsMOC1* |
| rs23935378 | Os06g0604500 | upstream | EF-Hand type domain containing protein. |  |
| rs26894569 | Os06g0656000 | intergenic | Hypothetical conserved gene. |  |
| rs26192911 | Os06g0643000 | intronic | Phox-like domain containing protein. |  |
| rs22686933 | Os09g0568000 | UTR3 | Single-stranded nucleic acid binding R3H domain containing protein. |  |
| rs1745509 | Os11g0137500 | exonic | Transcription factor TFE/TFIIEalpha, HTH domain domain containing protein. |  |
| rs16826302 | Os11g0480000 | exonic | NB-ARC domain containing protein. |  |

^a^SNP location identified by using CARMO database (http://bioinfo.sibs.ac.cn/carmo/SNP_Annotation.php).

^b^Candidate gene annotation collected from RAP-DB database (http://bioinfo.sibs.ac.cn/carmo/SNP_Annotation.php).

^c^Oryzabase (https://shigen.nig.ac.jp/rice/oryzabase/) used for gene symbol synonym.

**Supplementary Table S6 |** List of identified SNP and associated candidate gene regulating chalkiness percentage (CP) in rice.

| SNP | Candidate Genes | SNP location^a^ | Annotation^b^ | Gene Symbol Synonym^c^ |
| --- | --- | --- | --- | --- |
| rs4803005 | Os01g0190000 | upstream | Similar to oxidoreductase. |  |
| rs26502245 | Os02g0656100 | exonic | Conserved hypothetical protein. |  |
| rs35426658 | Os02g0824600 | intronic | Hypothetical conserved gene. |  |
| rs28088550 | Os04g0561000 | intergenic | Hypothetical protein. |  |
| rs154937 | Os05g0102600 | upstream | Zinc finger, RING/FYVE/PHD-type domain containing protein. | *OsRFPHC-11* |
| rs490822 | Os05g0108450 | intergenic | Hypothetical gene. |  |
| rs646158 | Os05g0112101 | intergenic | Epsin-like, N-terminal domain containing protein. |  |
| rs653823 | Os05g0112125 | intergenic | Hypothetical protein. |  |
| rs753549 | Os05g0114000 | intergenic | Similar to PRLI-interacting factor F (Fragment). |  |
| rs22670304 | Os07g0565300 | upstream | Bromodomain containing protein. |  |
| rs25558512 | Os07g0618800 | intergenic | Similar to caltractin. | *OsCML13* |
| rs25982235 | Os07g0627000 | intronic | Plastidial disproportionating enzyme1, alpha-1,4-D-glucanotransferase, Storage starch synthesis in rice endosperm | *OsDPE1* |
| rs26127356 | Os07g0629900 | upstream | Ribonuclease T2 domain containing protein. | *OsRNS8* |
| rs26290041 | Os07g0633400 | exonic | IQ calmodulin-binding region domain containing protein. |  |
| rs26314656 | Os07g0634100 | intronic | Conserved hypothetical protein. |  |
| rs26357917 | Os07g0634900 | downstream | Hypothetical conserved gene. | *OsMYB86* |
| rs26752914 | Os07g0642400 | intergenic | Similar to chromatin remodeling complex subunit. |  |
| rs26859579 | Os07g0644700 | intergenic | Hypothetical conserved gene. |  |
| rs14945421 | Os09g0416900 | intergenic | Armadillo-like helical domain containing protein. |  |
| rs16790346 | Os09g0449000 | intergenic | Hypothetical conserved gene. | *OsPTC1* |
| rs17097438 | Os09g0455000 | intergenic | Hypothetical protein. |  |

^a^SNP location identified by using CARMO database (http://bioinfo.sibs.ac.cn/carmo/SNP_Annotation.php).

^b^Candidate gene annotation collected from RAP-DB database (http://bioinfo.sibs.ac.cn/carmo/SNP_Annotation.php).

^c^Oryzabase (https://shigen.nig.ac.jp/rice/oryzabase/) used for gene symbol synonym.
